# Advances in Microfluidic Cochlea‐On‐A‐Chip

**DOI:** 10.1002/advs.202406077

**Published:** 2024-12-23

**Authors:** Tian Shen, Shanying Han, Weiwei He, Wen Yang, Xinghua Tang, Xiaolong Zhao, Xinghong Liu, Zhenhua Shao, Lin Cheng, Yu Zhao, Jiangang Fan

**Affiliations:** ^1^ Department of Otolaryngology‐Head & Neck Surgery West China Hospital Sichuan University Chengdu 610041 China; ^2^ Department of Otolaryngology Head and Neck Surgery Sichuan Provincial People's Hospital University of Electronic Science and Technology of China Chengdu 610072 China; ^3^ State Key Laboratory of Biotherapy and Cancer Center West China Hospital Sichuan University Chengdu Sichuan 610041 China; ^4^ Frontiers Medical Center Tianfu Jincheng Laboratory Chengdu 610212 China

**Keywords:** biomaterials, cochlea‐on‐a‐chip, disease modeling, drug testing, manufacturing strategies, microfluidic organ‐on‐a‐chip

## Abstract

The current understanding of the human auditory system has been primarily based on studies using animal and cellular models. Organoids have been used to simulate cochlear structures and replicate cochlear functions. However, the physical and chemical cues required to control the development of cochlear organoids accurately remain poorly understood, limiting research advances on cochlea‐on‐a‐chip systems. Consequently, the development of cochlea‐on‐a‐chip platforms that provide reliable preclinical testing grounds for studying inner ear developmental mechanisms and screening‐related therapeutic drugs has become a key focus for future cochlea‐on‐a‐chip technologies. In this review, the recent advancements in cochlea‐on‐a‐chip technology are summarized. First, an overview of cochlear anatomy and physiology is provided. Next, the latest breakthroughs are discussed in the 3D cultivation of inner ear organoids and explore the progress in microfluidic technologies for constructing cochlea‐on‐a‐chip systems. Finally, perspectives are presented on the current challenges and future directions for developing cochlea‐on‐a‐chip technology.

## Graphical abstract

This review systematically examines diverse cell sources for inner ear organoids and outlines stepwise induction protocols. Furthermore, it discusses current applications and prospective developments of cochlea‐on‐a‐chip technologies in areas such as deafness modeling, mechanistic studies, and drug evaluation, with particular focus on gene‐therapy drug testing, ototoxicity and otoprotective drug screening, auditory protection, and emerging areas such as auditory regeneration.

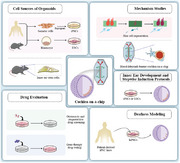

## 1 Introduction

Hearing impairment is a common sensory deficit, with global estimates suggesting ≈300 million cases.^[^ ^1^ ^]^ This condition arises from various etiological factors, including genetic predisposition, noise exposure, aging, and exposure to ototoxic agents. The global burden of hearing loss is substantial, manifesting in the number of affected individuals and its significant effects on quality of life, cognitive function, and economic productivity. Currently, cell‐based, molecular, or pharmacological therapies are lacking, and existing strategies primarily focus on hearing aids and cochlear implants, which do not restore hearing to normal levels. Consequently, cochlear organoids have emerged as a promising tool for developing effective therapies, as they allow for testing gene therapies for hereditary hearing loss and enable screening for drugs that promote hair cell regeneration after ototoxic damage.^[^ ^2^ ^]^ However, the inherent heterogeneity of cochlear organoids stemming from their complex and uncontrolled growth conditions limits their applicability in drug development.

To address these limitations, the organ‐on‐a‐chip (OOC) technology has been developed as a promising tool for simulating complex physiological environments. The foundational microfluidic technologies behind OOCs can be traced back to the early 1990s when Manz et al. introduced capillary electrophoresis on a chip, marking a significant advancement in miniaturized analytical devices.^[^ ^3^ ^]^ Microfluidic OOCs allow for the precise control of cellular microenvironments and enable the manipulation of external conditions, such as drug exposure and electrical stimulation, promoting tissue maturation and supporting the investigation of organ development and disease mechanisms in vitro.

**Figure 1:**
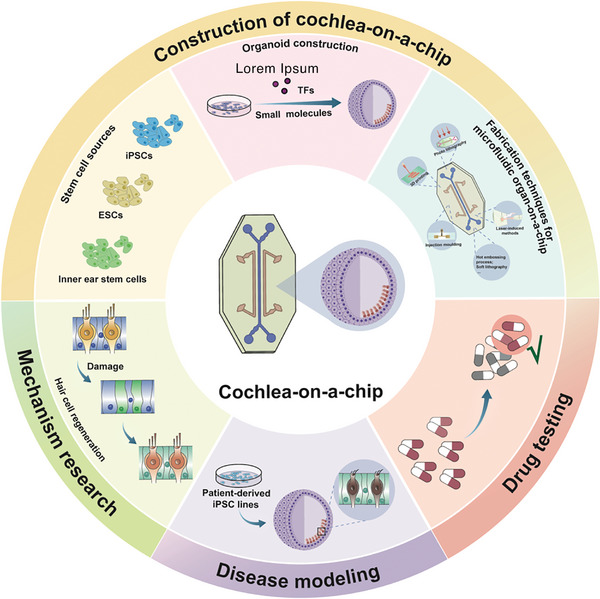
Schematic illustration of cochlea‐on‐a‐chip construction and application.

Moreover, recent legislative changes, including a modernization bill signed at the end of 2022, have underscored the U.S. Food and Drug Administration's endorsement of alternative animal testing methods for drug approval. This shift has accelerated the development of drug screening methodologies and highlighted the urgency of exploring fast and reliable candidate drug discovery approaches.^[^ ^4^ ^]^ Cochlea‐on‐a‐chip systems offer substantial advantages, including reduced costs and time associated with drug development, while simulating the structure and function of the human cochlea. Recent studies supporting the use of the cochlea‐on‐a‐chip in drug screening and disease modeling underscore its critical role in advancing therapeutic strategies for hearing loss.^[^ ^5^, ^6^, ^7^, ^8^ ^]^ In this review, we discuss the latest advancements and progress of cochlear organoids, the development of OOC technology, current applications of cochlea‐on‐a‐chip systems, and potential future research directions that may advance this field (**Figure** **1**).

## 2 Cochlea: Structure and Function in the Auditory System

**Figure 2:**
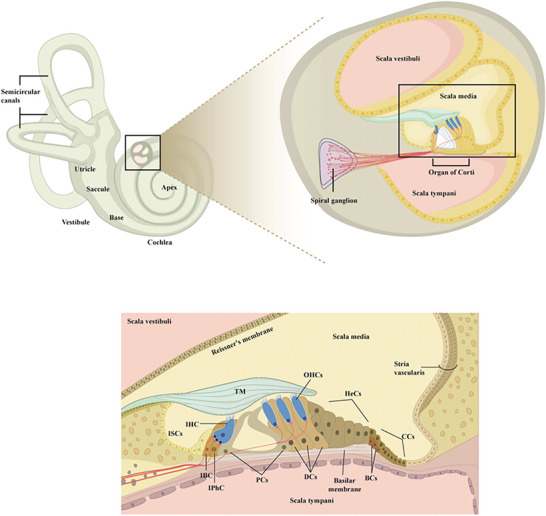
Schematic representation of the cochlea and the organ of Corti. The cochlea is divided into three fluid‐filled chambers, namely the scala vestibuli, tympani, and media, with the scala media containing K⁺‐rich endolymph. The organ of Corti, within the scala media, is the primary site for auditory mechanotransduction and is covered by the tectorial membrane (TM). It contains IHCs and OHCs, which are responsible for sensory transduction. Supporting cells, including Claudius cells (CCs), Deiters’ cells (DCs), Hensen's cells (HeCs), pillar cells (PCs), inner border cell (IBC), inner phalangeal cell (IPhC), inner sulcus cells (ISCs), and Boettcher cells (BCs).

The cochlea, a spirally coiled structure within the inner ear crucial for auditory function, is anatomically divided into three fluid‐filled chambers: the scala vestibuli, scala media (cochlear duct), and scala tympani, which are divided by Reissner's and basilar membranes. (**Figure** **2**). The scala vestibuli and tympani are filled with perilymph, whereas the scala media is filled with endolymph. The composition of endolymph is distinctive, characterized by a high potassium (K⁺) concentration (≈150 mm), low sodium (Na⁺) concentration (≈2 mM), and traces of calcium (Ca^2^⁺ at 20 µM), generating an endocochlear potential (EP) of ≈+80 mV compared with the perilymph and blood plasma.^[^ ^9^ ^]^

The stria vascularis, a metabolically active and richly vascularized tissue on the lateral wall of the scala media, plays a crucial role in secreting K⁺ into the endolymph and producing EP.^[^ ^10^ ^]^ The organ of Corti, situated on the basilar membrane in the scala media, houses auditory sensory cells, including three rows of outer hair cells (OHCs) and one of inner hair cells (IHCs), along with supporting cells that provide crucial structural assistance. Lgr5+ supporting cells, as well as those located in the greater epithelial ridge (GER) region of the cochlea, possess the capability to regenerate Myo7A‐positive hair cells.^[^ ^11^ ^]^

Wang et al. have shown that, when activated by damage, Lgr5+ supporting cells can produce hair cell‐like cells in the utricle of neonatal mice.^[^ ^12^ ^]^ This regeneration occurs via two mechanisms: 1) the direct transdifferentiation of supporting cells into hair cells and 2) mitotic regeneration, where supporting cells initially divide through mitosis before differentiating into hair cells. Each hair cell has a bundle of stereocilia‐actin‐rich projections arranged in a staircase‐like pattern. These stereocilia are interconnected by tip links, which are filamentous connections that extend from the tip of one stereocilium to the side of an adjacent, taller stereocilium. The tension in these tip links is critical for mechanotransduction.

The basilar membrane vibrates when sound waves induce changes in fluid pressure in the cochlea. This oscillation causes the stereocilia to bend toward the tallest ones, initiating a mechanotransduction cascade. This bending causes an increased influx of Ca^2^⁺ and K⁺ into the stereocilia, depolarizing the hair cell membrane.^[^ ^13^ ^]^ IHCs and OHCs play distinct and complementary roles in the auditory system. IHCs, ≈3500 in the human cochlea, are the primary sensory receptors for auditory stimuli that transduce mechanical vibrations induced by sound waves into electrochemical signals, releasing neurotransmitters at synaptic junctions with afferent auditory nerve fibers that transmit auditory information to the central nervous system. In contrast, OHCs exhibit unique electromotility properties in which changes in membrane potential cause alterations in cell length, enhancing the sensitivity and amplification of sound stimuli.

The complexity of the structure and function of the cochlea, which involves multiple distinct cell types with highly specialized roles, presents significant challenges for direct in vivo studies. Furthermore, the invasive nature of biopsy sampling limits the ability to examine the cochlear function in detail. To address these limitations, cochlear organoids represent a promising alternative by providing a 3D, human‐relevant model that accurately recapitulates cochlear development and cellular composition. These organoids enable more physiologically relevant studies on cochlear development, auditory disease modeling, and the molecular mechanisms underlying sensorineural hearing loss and offer a high‐throughput platform for drug discovery and testing, facilitating the evaluation of potential therapeutic agents. Moreover, cochlear organoids facilitate the investigation of regenerative approaches, such as hair cell regeneration, with potential applications in novel hearing loss treatments.

## 3 Advances in Inner Ear Organoids

**Figure 3:**
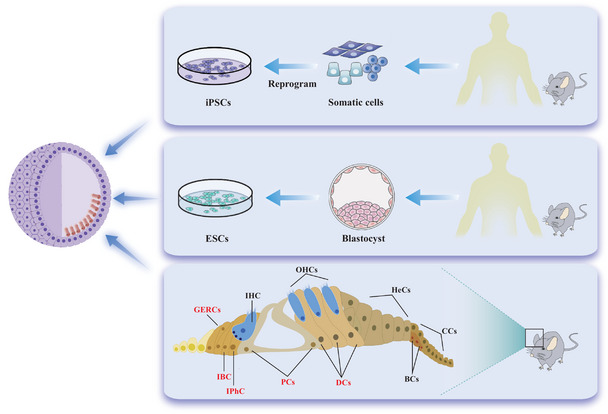
Schematic diagram of the cell origin of inner‐ear organoids. Inner‐ear organoids are generated from various stem cell sources, including embryonic stem cells (ESCs), induced pluripotent stem cells (iPSCs), and inner‐ear stem cells. Inner‐ear stem cells include Lgr5+ supporting cells, such as the third row of Deiters cells (DCs), inner phalangeal cells (IPhCs), pillar cells (PCs), inner border cells (IBCs), and greater epithelial ridge cells (GERCs).

Inner ear organoids can be generated from pluripotent stem cells, including embryonic stem cells (ESCs), induced pluripotent stem cells (iPSCs), and monopotent stem cells, such as inner ear stem cells (**Figure** **3**). Pluripotent stem cells can differentiate into various cell types across all three germ layers, including inner ear cells. These cells require the addition of specific cytokines, growth factors, or small molecules to facilitate differentiation toward inner ear lineages, necessitating the precise replication of various signaling pathways involved in inner ear organogenesis. In contrast, inner ear stem cells, which are monopotent, are committed to the inner ear lineage and possess limited differentiation potential; thus, they can be directly induced to form inner ear cells with fewer external factors, simplifying organoid formation.

### 3.1 Pluripotent Stem Cells

During the initial stages of embryogenesis, the ectoderm differentiates into two distinct regions: neuroectoderm and non‐neural ectoderm. The otic placode forms at the junction between the neuroectoderm and cranial non‐neural ectoderm in the otic‐epibranchial placode domain (OEPD). As development progresses, the otic placode undergoes invagination, forming the otic pit, which subsequently detaches from the surface ectoderm to form the otocyst (otic vesicle), the precursor to the entire inner ear.^[^ ^14^ ^]^ The early specification of otic cell fate during embryonic development is orchestrated by several coordinated signaling events, including fibroblast growth factors (FGFs), bone morphogenetic proteins (BMPs), WNT, sonic hedgehog (SHH), retinoic acid (RA), and transforming growth factor‐beta (TGF‐β).^[^ ^2^, ^15^, ^16^ ^]^ The downregulation of TGF‐β expression and increased FGF and BMP signaling facilitate the formation of the non‐neural ectoderm. Subsequently, diminished BMP signaling, combined with sustained FGF and WNT signaling, induces the formation of an otic OEPD within the non‐neural ectoderm. High WNT activity levels, originating from adjacent tissues, such as the neural tube and cranial mesenchyme, positively regulate components of the Notch signaling pathway, such as Jag1, which further enhances WNT signaling.^[^ ^17^ ^]^ Continuing WNT signaling, along with reduced FGF signaling, promotes the formation of the otic placode within the OEPD. In contrast, the lateral region of the OEPD, characterized by lower levels of WNT signaling and higher levels of FGF signaling, undergoes differentiation into epibranchial placodes. This differential signaling pattern guides the distinct specification of otic and epibranchial fates within the OEPD.^[^ ^18^ ^]^ Following the formation of the otocyst, several key signaling pathways, includingSHH, WNT, FGFs, andRA, orchestrate the complex morphogenesis of this structure, ultimately giving rise to highly specialized regions of the inner ear. SHH signaling, originating from the notochord and neural tube, is essential in ventral patterning, particularly in the development of the cochlea.^[^ ^19^ ^]^ In contrast, WNT and BMP signaling act as dorsalizing signals in the mouse otocyst.^[^ ^20^ ^]^ A balance between WNT and SHH signaling activities is essential for differentiating vestibular and cochlear structures. Existing biological cues have led to the development of an otic induction model in ESCs. Hashino et al. cultured human embryonic stem cells (hESCs; WA25 cell line) in an E8 medium for two days before transferring the cell aggregates to a medium containing a low concentration of Matrigel and FGF‐2, which promoted epithelialization and ectoderm differentiation on the surface of the aggregates. Subsequently, the combination of BMP4 and SB‐431542, a TGFβ inhibitor, facilitates the induction of non‐neural ectoderm by day 4. A mixture of FGF‐2 and LDN‐193189 (a BMP signaling inhibitor) has been used to stimulate the development of OEPD, which expressed markers including PAX8, NCAD, ECAD, SOX2, and TFAP2A, OEPD was formed on day 8.

Thereafter, WA25 cell aggregates were treated with CHIR99021 (CHIR), a GSK3β inhibitor known to activate the WNT signaling pathway. Nearly all aggregates between days 12 and 16 showed epithelial protrusions that expressed otic‐specific markers, such as JAG1, PAX2, PAX8, SOX2, and SOX10, thereby confirming their identity as otic cells.^[^ ^21^ ^]^

**Figure 4:**
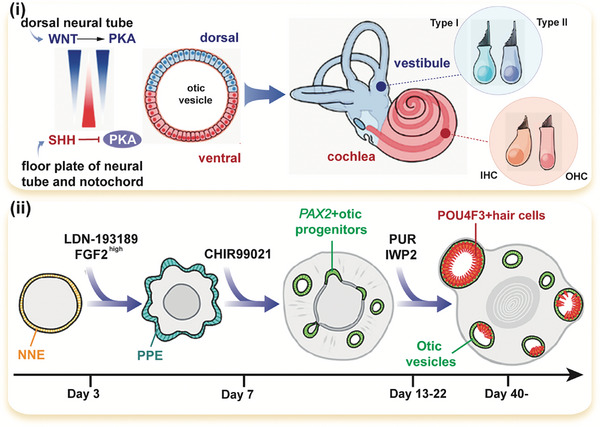
A schematic diagram illustrates the integration of established ventralization and dorsalization signals in mouse inner ear development (i) and the application of the sonic hedgehog (SHH) pathway agonist purmorphamine (PUR) and the WNT inhibitor IWP2 in the human inner ear organoid system (ii). Reproduced with permission.^[^ ^22^ ^]^ Copyright 2023, Cell Stem Cell.

Recently, Moore et al. improved the inner ear organoid culture system by implementing precise temporal adjustments of WNT and SHH signaling, effectively promoting ventral gene expression in otic progenitors (**Figure** **4**).^[^ ^22^ ^]^ Specifically, hESC‐derived organoids containing otic progenitors were cultured with the addition of the SHH pathway agonist purmorphamine (PUR) and the WNT inhibitor IWP2. The ventralized otic progenitors differentiated into intricately organized epithelia‐containing hair cells that exhibit marker expression, morphology, and functional characteristics of both the outer and inner cochlear hair cells.

In 2007, Takahashi et al. successfully reprogrammed adult human dermal fibroblasts into human induced pluripotent stem cells (hiPSCs) by introducing four key transcription factors: SOX2, KLF4, OCT4, and c‐MYC.^[^ ^23^ ^]^ These hiPSCs demonstrated properties similar to those of hESCs, including the ability to self‐renew and differentiate into various cell types representing the three germ layers. The advent of hiPSC technology has provided a transformative tool for investigating disease mechanisms, particularly genetic disorders. For instance, hiPSC lines generated with targeted gene knockouts or derived from somatic cells carrying disease‐specific mutations have been used to model inner ear dysfunction, offering insights into the molecular basis of these disorders.^[^ ^24^, ^25^, ^26^, ^27^ ^]^ A significant advantage of hiPSCs is their derivation without requiring surplus embryos, which addresses the ethical concerns associated with hESC research. Moreover, hiPSC lines can be personalized, stored in biobanks, and utilized for further research on individualized disease mechanisms, facilitating precision medicine. Several reviews have summarized the various induction protocols developed to differentiate hiPSCs into hair cell (HC)‐like cells.^[^ ^28^, ^29^ ^]^ To date, nearly all studies involving hiPSCs to induce inner ear organogenesis have relied on the stepwise induction approach established by Koehler et al.^[^ ^21^, ^30^, ^31^ ^]^ However, despite these advances, several studies have demonstrated significant genetic background variations among different hiPSC lines, leading to considerable variability in the directed differentiation process.^[^ ^32^, ^33^ ^]^ For instance, studies using murine iPSC (miPSC) lines have demonstrated that the optimal timing for FGF‐2/LDN‐193189 treatment during the stepwise induction of inner ear organoids varies across different cell lines.^[^ ^34^ ^]^ In contrast, genetically engineered ESCs, when free from off‐target mutations, exhibit a homogeneous genetic background. This uniformity facilitates the consistent generation of inner ear organoids and allows for better control over genetic background variability when comparing mutant and wild‐type PSC‐derived cells or tissues.

### 3.2 Inner Ear Stem Cells

Inner ear stem cells are a crucial cellular source for inner ear organoids. Lgr5 is a well‐established marker for cochlear progenitor cells within the inner ear.^[^ ^35^ ^]^ Lgr5+ cells have been identified in inner phalangeal cells, the third row of Deiters' cells, inner pillar cells, and inner border cells. (Figure 3).^[^ ^36^, ^37^ ^]^ Moreover, the capacity of subpopulations of inner ear stem cells expressing WNT pathway‐related genes, such as Axin2, Frizzled9, and Lgr6, to regenerate hair cells has been confirmed.^[^ ^38^, ^39^, ^40^, ^41^ ^]^ Additionally, the stem cells in the GER region of the cochlea exhibit similar regenerative capabilities for functional hair cell regeneration.^[^ ^42^ ^]^ Inner ear organoids can be generated from Lgr5+ inner ear stem cells using a small‐molecule approach that promotes their expansion and differentiation.

This process involves culturing the stem cells with growth factors, including β‐FGF, EGF, and IGF‐1, in combination with a GSK3β inhibitor to activate the Wnt signaling pathway and an HDAC inhibitor to modulate Notch signaling.^[^ ^43^ ^]^

### 3.3 Construction of Inner Ear Organoids

The abilities of 2D and 3D culture systems to model tissue complexities significantly differ. In 2D cultures, cells are grown on flat surfaces, which limits their ability to replicate the intricate cell–cell and cell–extracellular matrix (ECM) interactions observed in vivo, resulting in immature cell types.^[^ ^44^, ^45^ ^]^ Meanwhile, 3D cell culture systems, widely used for stem cell spheroid formation, rely on the fundamental concept of self‐assembly, where cells organize into spheroids when unable to adhere to the substrate. This self‐organization fosters tight cell‐cell and cell‐ECM interactions, significantly influencing stem cell behavior, such as viability, response to stimuli, and protein secretion, which differ markedly from traditional 2D cultures.^[^ ^46^ ^]^

To optimize 3D spheroid formation, various platforms, such as hanging drops, spinner flasks, hydrogels, and microfluidic chips, have been developed. These systems can be classified as scaffold‐based or scaffold‐free. Scaffold‐free methods rely on cell‐cell connectivity without external biomaterials, while scaffold‐based systems employ materials with tunable properties, such as porosity and stiffness, to mimic the tissue microenvironment, supporting cell adhesion, migration, and growth.^[^ ^46^ ^]^ Matrigel is commonly used in 3D cell culture systems as it effectively simulates the extracellular matrix. However, there are several notable limitations. Although it is generally characterized, its composition showed batch‐to‐batch variability, potentially compromising the reproducibility of experimental results.^[^ ^47^ ^]^ Additionally, Matrigel lacks precise control over mechanical properties, such as stiffness and porosity, which are critical for replicating the specific microenvironments required for different tissue types. To address these limitations, Zhang et al. incorporated a Ti3C2Tx MXene nanomaterial into Matrigel to create a composite matrix (MXene‐Matrigel) with improved mechanical properties and retained biocompatibility.^[^ ^48^ ^]^ The results indicated that MXene‐Matrigel promoted the maturation and differentiation of functional organoid hair cells, demonstrating superior electrophysiological properties to those of Matrigel. Furthermore, MXene‐Matrigel facilitates efficient synapse formation between ganglion neurons and hair cells in a coculture system, thus overcoming the limitations of traditional Matrigel‐based systems.

GelMA hydrogel is also used in inner ear studies owing to its excellent biocompatibility and controllable mechanical properties. Moreover, the composite hydrogel system based on GelMA can generate the matrix network required by specific tissues by altering the doped materials or biological components.^[^ ^49^ ^]^ In a typical experiment, GelMA hydrogel and PEDOT:PSS were combined, and this combination was proven to have potential as a coating for the intracochlear electrode used in cochlear implants. The sustainable release of the drug mixtures (RepSox, CHIR, and DAPT) encapsulated in the composite conductive hydrogel had dual effects. The drug delivery system based on the conductive hydrogel promoted the proliferation of cochlear stem cells and the trans‐differentiation of supporting cells into hair cells. The sustained release of drugs has a protective effect on residual hair cells by reducing programmed cell death, including apoptosis and ferroptosis.^[^ ^50^ ^]^ A 3D system consisting of GelMA hydrogel with controllable mechanical properties and HA‐Arg‐Gly Asp peptide that promotes the adhesion of cochlear progenitor cells (CPCs) was created to cultivate CPC‐derived organoid cells.^[^ ^51^ ^]^

GelMA is a gelatin‐derived biomaterial that undergoes photoinitiated radical polymerization to form covalently cross‐linked hydrogels that incorporate RGD sequences and matrix metalloproteinase target sites for enhanced cell attachment and remodeling; this offers superior solubility, reduced antigenicity, and customizable mechanical properties, making it suitable for tissue engineering applications.^[^ ^52^ ^]^ Recently, GelMA has been applied as a stiffness‐adjustable hydrogel system to regulate cochlear organoid development by modulating ECM mechanical forces, which drive cochlear progenitor cell proliferation and differentiation into sensory hair cells through integrin α3/F‐actin cytoskeleton/YAP and Ca^2^^+^/ERK1/2/KLF2 signaling pathways.^[^ ^51^ ^]^

Physical and chemical signals are fundamental to organ development and functional remodeling. Despite significant advancements in creating various 3D scaffolds that simulate the in situ microenvironment for organoid systems, these scaffolds often lack the precise control required to fully replicate organ functionality, which limits their clinical translation.^[^ ^53^ ^]^ OOC technology offers a solution by providing a high‐throughput, high‐fidelity platform that integrates cultivation, testing, and analysis to better reproduce tissue structure and function. This technology automates and standardizes the process and offers more precise control over environmental conditions than traditional 3D scaffolds. Recent studies have reported several human OOC models focused on disease modeling, utilizing microfluidic technology to closely mimic organ systems.^[^ ^54^ ^]^ Established models, which include the liver, heart, intestine, and lung‐on‐a‐chip systems, have been used to simulate physiological conditions for drug testing and disease research.^[^ ^55^, ^56^ ^]^ However, research on cochlear function and its in vitro replication is in its early stages. The physical and chemical cues required to construct functional cochlear organoids are not yet fully understood, and the development of cochlea‐on‐a‐chip models is limited. Consequently, the field of inner ear organoids and cochlea‐on‐a‐chip technology remains in its nascent phase yet has significant potential for further exploration. In the following sections, we provide a comprehensive overview of the fabrication techniques for manufacturing microfluidic OOCs, along with an assessment of the current state of cochlea‐on‐a‐chip research and potential future directions for this field.

## 4 Fabrication Techniques for Manufacturing Microfluidic OOC Devices

OOC technology has attracted attention in biomedical studies owing to its ability to mimic human physiology in vitro.^[^ ^57^ ^]^ OOC components include cell culture modules, microfluidic systems, external stimuli, and biosensors. OOC microfluidic devices are currently the most suitable technology for replicating human tissues and organs, potentially facilitating further clinical studies. Microfabrication is a process in which various techniques are used to pattern substrate materials to create the desired shapes. Regarding microscopic textures, there are several benefits to the unique properties of fluids in a microenvironment. The laminar flow, concentration gradient, and high surface area‐to‐volume ratio of the channels replicate the structures and functions observed in biological systems.^[^ ^58^ ^]^ Microfluidics is a relatively new field in OOC devices. Various biomaterials and microfabrication techniques have been developed that cover various areas of biological studies. Different biomaterials and microfabrication techniques have been used to create OOC systems, each with unique advantages and disadvantages.^[^ ^59^ ^]^

### 4.1 Commonly Selected Biomaterials in OOC Systems

The selection of biomaterials is vital in the construction of OOC systems, as they form the basis for tissue fabrication and play a key role in determining the overall functionality of the chip system.^[^ ^61^ ^]^ Biomaterials can be categorized into natural and synthetic polymers based on their origin. Those selected for OOC systems are generally non‐toxic to cells, breathable (in certain cases), i.e., promote cell respiration, and optically transparent; however, they differ in biocompatibility, biodegradability, mechanical properties, manufacturing complexity, and cost.^[^ ^59^, ^62^ ^]^ Given the complexity and diversity of human tissues and organs, OOC systems must be carefully tailored with specific biomaterials or combinations to effectively simulate different tissue types.^[^ ^60^ ^]^ With extensive studies and increasing demand for simulating complex organ functions and structures, various emerging biomaterials have been developed for OOC manufacturing.^[^ ^63^, ^64^ ^]^

Natural polymers from extracellular matrix components, mammals, insects, and plants are highly biocompatible. They promote cell adhesion, proliferation, rheological dynamics, and mechanical stimulation and are essential in constructing complex organ and tissue microenvironments and simulating specific physiological functions.^[^ ^61^, ^65^ ^]^ Common natural polymers include gelatin, collagen, hyaluronic acid, Matrigel TM, fibrin, chitosan, silk fibroin, and alginate.^[^ ^62^ ^]^ In otological research, natural polymers have been applied. For instance, Yu et al. developed a minimally invasive middle ear delivery system using a brain‐derived neurotrophic factor (BDNF)‐poly‐(dl‐lactic acid‐co‐glycolic acid) (PLGA)‐loaded hydrogel, which effectively reversed cochlear synaptopathy and restored hearing function in a noise‐induced hearing loss (NIHL) mouse model, highlighting the potential of targeted synaptic repair for treating sensorineural deafness.^[^ ^66^ ^]^ Furthermore, Matrigel™ is one of the most widely used natural polymers in the cultivation of cochlear organoids, owing to its ability to closely mimic the native extracellular matrix environment.

Synthetic polymers have been designed to accurately regulate their physical and chemical characteristics to meet the requirements of different OOCs and reduce costs.^[^ ^61^ ^]^ These polymers have been used due to the easy adjustment of their porosity, surface roughness, and mechanical properties. However, their limitations include the absorption of small hydrophobic molecules and insolubility in organic solvents.^[^ ^67^ ^]^

Poly(dimethylsiloxane) (PDMS) is a widely used synthetic polymer chip material with optical transparency, biocompatibility, gas permeation properties, and flexibility. Porous PDMS membranes exhibit high porosity and low stiffness, highlighting the significance of material selection in creating biomimetic microenvironments for cellular studies.^[^ ^68^, ^69^ ^]^ PDMS membranes have been used to simulate organs, such as the liver, kidney, and blood vessels.^[^ ^70^ ^]^ In cochlear studies, most synthetic polymers are used to monitor and mimic human cochlear functions. In Stieglitz's study, PDMS was used as a material for optical probes of the cochlea owing to its flexibility. Furthermore, the authors investigated the long‐term usability of PDMS regarding transmission and refractiveness.^[^ ^74^ ^]^ Adding polyethylene glycol has been shown to increase the tectorial membrane (TM) viscosity and broaden the tuning range of resonance‐based TM models.^76^Similar to PDMS, polycarbonate (PC), a hard material, is usually used as a tissue‐bearing porous membrane component.^[^ ^71^ ^]^ Polyolefins, such as cyclic olefin polymers, cyclic olefin copolymers, and polymethylmethacrylate (PMMA), are also promising for OOC manufacturing since they are generally low‐cost, biocompatible, lack small‐molecule absorption, and have good mechanical strength.^[^ ^72^ ^]^

Similarly, aliphatic polyesters, such as polylactic acid (PLA) and poly (ε‐caprolactone) (PCL), have low absorption, low autofluorescence, and sustainable characteristics, making them a suitable plastic alternative in OOC applications.^[^ ^73^ ^]^ However, the application of these biomaterials in cochlea‐on‐a‐chip systems remains to be fully developed. Although existing natural and synthetic polymers have demonstrated good biocompatibility and tunability in other OOC systems, their optimization for the unique cochlear microenvironment remains warranted.

### 4.2 Device Fabrication Techniques for Manufacturing Microfluidic OOC Devices

Microfluidic systems, an essential design factor in developing OOCs, provide necessary survival support for tissue construction, cell culture, and microenvironment cycling to accurately manipulate physiological and chemical parameters. Various microfabrication techniques have been developed for manufacturing microfluidic OOC (**Table** **1**).

**Table 1:** Pros and cons of fabrication technique and their application in different materials.

Fabrication Technique	Advantages	Limitations	Materials and Application
Photo lithography	High precision; Comparatively fast; Used on different materials	High initial tooling and machinery costs; Low throughput; Inability to allow gas to permeate	Resin^[^ ^76^ ^]^
			Elastomers^[^ ^77^ ^]^
			Cyclic olefin polymers^[^ ^78^ ^]^
Soft lithography	Capable of mass production Cost‐effective; Easy to use; High throughput	Low bio‐resistance; Requires dedicated equipment; Familiar with technical manual operation	PDMS^[^ ^79^ ^]^
			Liquid metal^[^ ^80^ ^]^
			Polystyrene^[^ ^81^ ^]^
Injection moulding	High repeatability and reliability; Great for large‐scale production; Complex geometries	High initial tooling and machinery costs; Requires high technical knowledge; Limited materials available	Polystyrene^[^ ^82^ ^]^
			Polymethylmethacrylate^[^ ^83^ ^]^
Hot embossing process	Cost‐effective; High efficiency; Capable of mass replication	Require the optimal process parameters; Specific production materials	Polymethylmethacrylate^[^ ^84^ ^]^
			Cyclic olefin copolymer^[^ ^85^ ^]^
			Polystyrene^[^ ^86^ ^]^
Laser‐induced methods	Excellent accuracy and precision; Short development time	Require high technical knowledge; High costs	Paper^[^ ^87^ ^]^
			Monocrystalline silicon^[^ ^88^ ^]^
3D printing	Versatility; Flexible designing and modelling; Compatible with various materials; Rapid prototyping; Cost‐effective	Low resolution; Restricted sizes; Slower production	PDMS^[^ ^89^ ^]^
			Glass^[^ ^90^ ^]^
			Poly(ethylene glycol)^[^ ^91^ ^]^
			Resin^[^ ^92^ ^]^
			Natural biopolymers^[^ ^93^ ^]^

PDMS: poly(dimethylsiloxane).

Lithography is used to create OOCs with features on the nanometer or micrometer scale, consistent with the dimensional shape and size of cell interactions in their natural environment.^[^ ^94^ ^]^

Currently, OOCs primarily use photolithography and soft photolithography. Photolithography involves the creation of 3D micropatterns on a silicon substrate through selective exposure to light, primarily UV light, at wavelengths of 250–435 nm. The photoresist layer was applied to a silicon wafer via spin coating or lamination and subsequently exposed to UV light to alter and solidify their properties. Unexposed regions were baked and washed in a chemical bath. Using the exposure method, lithography can be divided into mask lithography (in which UV light is applied to the wafer through photomasks) and maskless photolithography (in which UV light is applied to the wafer through direct laser exposure). Similar to traditional technology, soft lithography is based on conventional photolithography and uses a photoresist and UV light to produce a 3D micropattern to build a mold. The mold covered with the crosslinking agent was mixed with a synthetic polymer and cured at a specific temperature to create the final device. Among synthetic polymers, PDMS is the most commonly used owing to its excellent biomedical properties and oxygen permeability. (Soft) lithography is a cost‐effective and versatile technique commonly used in fabricating microfluidic devices; hence, it is a popular choice in the industry.^[^ ^95^, ^96^ ^]^ Kasi et al. recently introduced a rapid and cleanroom‐free microfabrication method for OOC devices using maskless photolithography.^[^ ^95^ ^]^ In addition to traditional photolithography, in‐situ photolithography has been used to coat the interior of microfluidic channels and create monolayers of hydrogels in OOC devices.^[^ ^96^ ^]^

Injection molding has long been used for large‐scale polymer manufacturing and includes four stages: 1) Injection: the liquid plastic is injected into a well‐established mold; 2) holding and packing; 3) cooling and solidification; 4) mold opening and part ejection.^[^ ^97^ ^]^ Controlling suitable materials, nanoscale geometries, and the appropriate injection rate is crucial to ensure high production quality. Injection molding is suitable for producing microscale OOC devices owing to its efficiency and scalability. Similarly, injection molding in OOC technology has been highlighted in several studies. Lee et al. fabricated a tubule‐on‐a‐chip with HUVEC and RPTEC co‐cultures using injection‐molded polycarbonate chips.^[^ ^98^ ^]^ Yu et al. introduced a perfusable microvascularized 3D tissue array created using injection molding, which enabled high‐throughput vascular phenotypic screening.^[^ ^99^ ^]^

Hot embossing is suitable for fabricating microfluidic devices, particularly microchannels. First, a master mold is designed, fabricated, and pressed into a polymeric solution to create a new device. To achieve the highest precision, the material thickness, applied pressure, and heating temperature must be precisely controlled. Despite these challenges, hot embossing offers numerous benefits, including low cost and excellent structural precision, making it the preferred method to other techniques. In cardiac tissue engineering and heart OOC technologies, hot embossing has been used to create microfluidic chips; this demonstrates the suitability of this technique for cardiac applications.^[^ ^100^ ^]^ Furthermore, the combination of hot embossing and 3D printing has been explored to scale up the production of OOC devices.^[^ ^101^ ^]^ The ability of this method to replicate intricate structures and create complex microchannels makes it valuable for mimicking physiological conditions in vitro.

Laser‐induced methods rely on lasers to construct complex micropatterns on substrates. These methods allow for constructing patterns with varying depths and widths based on computer designs. Recently, laser‐related methods have been commercialized for microfluidic devices owing to their non‐contact carving and excellent resolution. Researchers have explored innovative fabrication techniques to enhance and expand the versatility of laser‐induced technologies on OOC platforms. For example, Shin et al. demonstrated the monolithic digital patterning of PDMS using successive laser pyrolysis and developed distinct microfluidic devices with elaborate channel architectures, revealing the versatility of laser‐based methods for creating complex microfluidic devices for various technological applications.^[^ ^102^ ^]^ Furthermore, Perrone et al. explored the fabrication of quartz microchannels and microholes using pulsed CO_2_‐laser ablation and highlighted their potential for constructing biological models and biomedical applications.^[^ ^103^ ^]^

3D printing technology has been a valuable tool for studying cellular processes, designing customized medications, and constructing sophisticated OOC platforms. This process involves layer‐by‐layer fabrication, in which materials are laid from the bottom layers to the top. Different types of 3D printing technologies include extrusion‐based, plaster‐based, inkjet printing, and light‐polymerized technology.^[^ ^104^, ^105^ ^]^ In extrusion‐based technology, a thermoplastic material is fed into a heated extrusion head, which melts and solidifies as it extrudes. Plaster‐based inkjet printing has been successfully used to create complex shapes of polymers, metals, jet‐ink materials, and ceramic objects. Light‐polymerized 3D printing uses laser beams to cure photosensitive thermoset polymeric materials and sculpt the polymers by modifying the beam direction. Advancements in the high‐resolution and low‐cost fabrication capabilities of 3D printing technology have simplified the fabrication of OOC devices. In addition, 3D printing offers more benefits, such as the freedom of complex geometries, relatively simple manufacturing conditions, and less waste of raw materials. Bioprinting, an extension of 3D printing technology, is the latest innovation in microfluidic chip development for OOCs that involves placing live cells and biomaterials into defined spatial scaffolds and structures.

These technologies are essential for manufacturing organ analogs and living tissues in various fields, such as complex cellular signaling, biocompatible organ surrogates, pharmacokinetics, and novel drug development. Over the past decades, although conventional manufacturing techniques, particularly lithography, have been used to manufacture OOC, they require complicated and time‐consuming procedures and a cleanroom environment. Compared to other techniques, 3D printing techniques are simple, cost‐effective, robust, and allow the mass manufacturing of OOC devices. 3D printing can be used to accurately control the spatial distribution of biomaterials and provide desired tissue‐specific functions and 3D cellular arrangements on a chip. In the future, 3D printing may provide high‐throughput analysis and high‐precision and resolution manufacturing methods for microfluidic cochlea‐on‐a‐chip.

## 5 Microfluidic Cochlea‐On‐A‐Chip System

The cochlear organoid cell sources discussed above, including pluripotent and inner ear stem cells, can theoretically be incorporated into the microfluidic cochlea‐on‐a‐chip system.^[^ ^5^ ^]^ This platform offers a significant advantage owing to its precise regulation of the microenvironment surrounding the cultured cells. In contrast, traditional inner ear organoids cultured within 3D matrices have inherent limitations in controlling and manipulating key environmental parameters, restricting their capacity for fine‐tuned physiological simulations.

This precision is particularly crucial for mechanistic studies, drug screening, and disease modeling as it allows for maintaining consistent and reproducible culture conditions, a difficult‐to‐achieve outcome with traditional organoid models. Consequently, the cochlea‐on‐a‐chip platform represents a transformative tool for advancing the understanding of cochlear physiology and pathophysiology and accelerating the development of therapeutic interventions for inner ear disorders.

### 5.1 Applications in Mechanistic Studies

The development and physiological mechanisms of the cochlea pose challenges due to complex cellular interactions and signaling pathways. However, cochlea‐on‐a‐chip technology has expanded our understanding of this area. By replicating the cochlear microenvironment and enabling the precise monitoring of cellular behavior, this innovative platform has facilitated the exploration of the fundamental mechanisms underlying cochlear development, which are difficult to investigate using traditional in vitro models.

Cochlea‐on‐a‐chip technology provides novel insights into cochlear development and its underlying physiological mechanisms.

For example, a cochlea‐on‐a‐chip platform integrates cochlear organotypic cultures (COCs) with high‐ATP‐sensitivity biosensors (ATP‐WCBs) in a custom‐designed transparent microfluidic chamber.^[^ ^5^ ^]^ Using dual multiphoton Ca^2+^ imaging, researchers have simultaneously monitored intercellular Ca^2+^ waves in the GER of COCs and ATP‐dependent Ca^2+^ responses in biosensors. The inhibition of Ca^2+^ signals with ATP diphosphohydrolase (apyrase) and the absence of connexin 30 with downregulated connexin 26 in Gjb6^−/−^ COCs confirmed that the release of ATP through connexin hemichannels drives spontaneous Ca^2+^ signaling in the developing cochlea's non‐sensory cells.

Neuron‐ and Schwann cell‐like cells were successfully produced in a previous study using a cochlea‐on‐a‐chip derived from mouse ESCs.^[^ ^6^ ^]^ The onset of myelination was neuron‐like and Schwann cell‐like cells interacted within the microfluidic device. This platform enabled real‐time monitoring of these interactions. Blocking macrophage migration inhibitory factor (MIF) on the Schwann cell‐like side significantly reduces neurite outgrowth, demonstrating the role of MIF in neuronal guidance. These findings have potential implications for improving cochlear implant efficacy since MIF‐expressing Schwann cell‐like cells have been used to coat cochlear implants, facilitating the directional growth of spiral ganglion neurons (SGNs) toward the implant. This indicates that the cochlea‐on‐a‐chip can be used to replicate developmental processes and study inner ear cell interactions in controlled environments, which are difficult to achieve using traditional in vitro models.

### 5.2 Deafness Modeling and Gene Therapy Drug Testing

The cochlear organoid model has been successfully used to study hearing loss caused by mutations in genes such as *Tmprss3*, *SLA26A4*, *Gib2*, *MYO7A*, and *MYO15A*.^[^ ^24^, ^25^, ^26^, ^27^ ^]^ However, owing to operational and structural limitations, traditional organoids are less suitable for large‐scale drug screening. For genetic‐attributed hearing loss, in which the effects of different mutations on the auditory system significantly vary, the development of personalized therapeutic models is crucial.

The cochlea‐on‐a‐chip platform offers distinct advantages for high‐throughput screening and is particularly valuable for developing gene therapies for hereditary hearing loss. Using patient‐derived iPSCs, personalized cochlea‐on‐a‐chip models that target specific genetic mutations can be created. These models enable simultaneous testing and evaluation of various gene therapy drugs, allowing for the rapid identification of potential treatments. Furthermore, different cochlea‐on‐a‐chip platforms can be developed for various mutation sites within the same gene to facilitate the precise testing and evaluation of one‐ or multiple‐gene therapy drugs.

### 5.3 Ototoxicity and Hearing Protection Drug Testing

The intricate 3D structure and microenvironment of the human cochlea and the difficulty in obtaining human cochlear tissue samples pose significant challenges for in vivo research on ototoxicity and drug protection mechanisms. Consequently, traditional drug studies on ototoxicity and hearing protection have relied predominantly on animal models, particularly mice. However, fundamental anatomical, molecular, and immunological differences between mouse and human cochleae limit the translational value of these models.^[^ ^106^ ^]^

The cochlea‐on‐a‐chip platform is a promising tool for screening ototoxic and hearing‐protective drugs. Although studies using hiPSC‐ and hESC‐derived cochlea‐on‐a‐chip systems remain limited, these platforms hold great potential for high‐throughput drug testing, offering an in vitro model that more accurately replicates human cochlear physiology while providing a controlled environment for drug evaluation.

**Figure 5:**
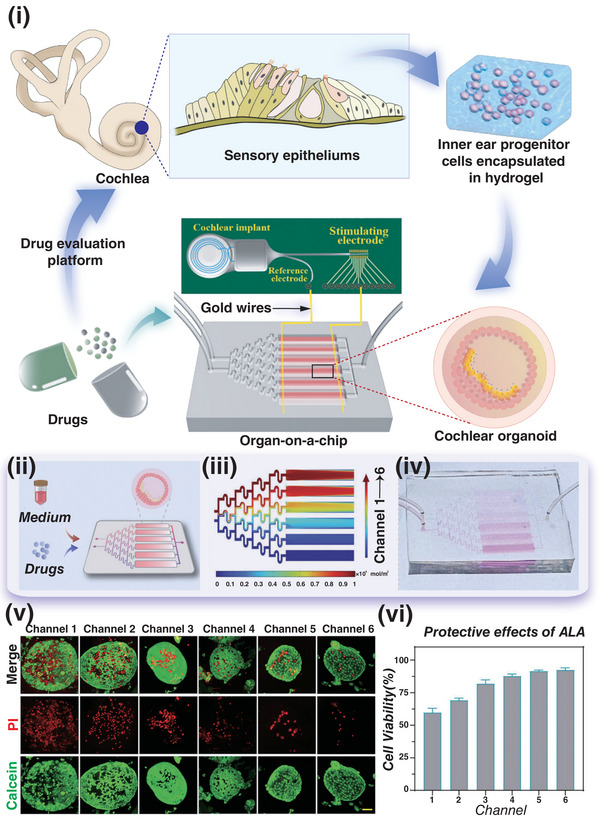
Electroacoustic responsive cochlea‐on‐a‐chip platform. i) A schematic diagram of the cochlea‐on‐a‐chip construction process and its application in high‐throughput drug screening on the integrated platform. ii) Schematic diagram of drug evaluation based on the cochlea‐on‐a‐chip. iii) Use of a gradient generator to produce drug concentration gradients on the cochlea‐ on‐a‐chip. iv) Photographs of constructed cochlea‐on‐a‐chip with different drug concentration gradients. v) Live/dead fluorescence staining images of cochlear organoids located in the culture zone on the chip, which were used to evaluate the protective effect of varying concentrations of alpha‐lipoic acid on cochlear organoids treated with cisplatin. Scale bar = 50 µm. vi) The relative cell viability of cochlear organoids obtained from live/dead fluorescence staining data (*n* = 4). Reproduced with permission.^[^ ^8^ ^]^ Copyright 2024, Advanced Materials.

Hu et al. recently demonstrated the potential of using cochlea‐on‐a‐chip in evaluating otoprotective drugs (**Figure** **5**).^[^ ^8^ ^]^ The authors integrated mouse inner ear stem cell‐derived cochlear organoids with a conductive hydrogel biohybrid system and cochlear implant electroacoustic stimulation to create a functional cochlea‐on‐a‐chip platform. This microfluidic‐based device, equipped with culture chambers and a concentration gradient generator, can enable dynamic and high‐throughput evaluation of otoprotective drugs, assess ototoxicity, and facilitate studies on hair cell regeneration therapies.

### 5.4 Blood‐Labyrinth Barrier Cochlea‐On‐A‐Chip Model

The blood‐labyrinth barrier (BLB) is crucial in regulating the exchange of substances between the blood and cochlear fluid, protecting the inner ear from potentially harmful substances while maintaining ionic homeostasis essential for hearing.^[^ ^107^ ^]^ Nevertheless, the structure and function of the BLB remain poorly understood, particularly in pathological conditions such as inflammation, ototoxicity, and genetic disorders.

A cochlea‐on‐a‐chip platform that models the BLB could provide valuable insights into drug permeability, ototoxicity mechanisms, and potential therapeutic interventions. This platform would be particularly beneficial for high‐throughput drug screening, enabling researchers to determine how various compounds, such as otoprotective agents or ototoxic drugs, interact with BLB.

In a recent study, BLB was isolated from post‐mortem human tissue, and a culture system for endothelial cells and pericytes was successfully established on a BLB chip^[^ ^7^ ^]^. This innovative model was used to measure the permeability of two pharmacologically relevant compounds: midazolam (molecular weight: 325.78 Da) and daptomycin (molecular weight: 1.6 kDa). These findings confirm that the BLB chip accurately replicated the permeability and integrity of the human stria vascularis capillaries. This model is a valuable tool for investigating the physiological and pathological mechanisms of BLB and evaluating drug delivery systems targeting the inner ear. By manipulating variables, such as drug concentration, exposure duration, and inflammatory stimuli, researchers can gain deeper insights into the role of BLB in protecting or compromising cochlear function. Therefore, developing a BLB cochlea‐on‐a‐chip model is a promising and valuable direction for future studies.

## 6 Conclusion

Over the past decade, significant progress has been made in the construction and application of cochlear organoids, offering new opportunities for research and therapeutic interventions. The development of the cochlea‐on‐a‐chip technology has enabled the creation of more sophisticated cochlear organoids, which are recommended in mechanistic studies, disease modeling, and drug screening.

Further investigations into the signaling pathways involved in cochlear development under physiological conditions remain warranted to enhance the development of more physiologically relevant microfluidic cochlea‐on‐a‐chip models. These systems currently face challenges, such as high manufacturing costs, complex operational procedures, and a lack of standardized protocols, which limit their application in preclinical settings.

Advanced micro/nanomanufacturing technologies, particularly 3D bioprinting technology, should be considered for constructing cochlea‐on‐a‐chip systems. This innovative approach can be used to mitigate high‐cost limitations associated with traditional lithography techniques, enhancing the accessibility and practicality of organoid production. Future studies should integrate the latest microfluidic technologies to design new cochlea‐on‐a‐chip models and foster the development of novel integrated systems. Additionally, construction technologies should be integrated into the cochlea‐on‐a‐chip system to replicate refined cochlear microstructures and in vitro disease modeling applications.

## Conflict of Interest

The authors declare no conflict of interest.
